# Progressive Cartilage Degeneration After Anterior Cruciate Ligament Reconstruction: Longitudinal Evidence From the Swedish Knee Ligament Registry

**DOI:** 10.1177/03635465261438145

**Published:** 2026-04-20

**Authors:** Piero Agostinone, Iacopo Romandini, Pierre Rotzius, Stefano Zaffagnini, Magnus Forssblad, Alexander Sandon

**Affiliations:** * Dipartimento di Scienze Biomediche e Neuromotorie, University of Bologna, Bologna, Italy; † Clinica Ortopedica e Traumatologica II, Istituto Ortopedico Rizzoli, Bologna, Italy; ‡ Södersjukhuset/Karolinska Institute, Stockholm, Sweden; § Department of Molecular Medicine and Surgery, Stockholm Sports Trauma Research Center, Karolinska Institute, Stockholm, Sweden; Investigation performed at Karolinska Institute, Stockholm, Sweden

**Keywords:** knee, articular cartilage, ACL, epidemiology

## Abstract

**Background:**

Chondral lesions and subsequent knee osteoarthritis often occur after anterior cruciate ligament (ACL) injuries, and although ACL reconstruction (ACLR) is common, cartilage degeneration remains more prevalent than in the general population, with inconsistent evidence on whether surgery offers a protective effect.

**Purpose/Hypothesis:**

This study aimed to evaluate the progression of cartilage damage after ACLR using second-look arthroscopy in patients who underwent both primary and revision procedures and to identify risk factors for cartilage degeneration. It was hypothesized that chondral lesions would progress between primary and revision surgery, primarily influenced by characteristics of the initial injury and primary surgery.

**Study Design:**

Cohort study; Level of evidence, 3.

**Methods:**

A retrospective cohort study was conducted using prospectively collected data from the Swedish Knee Ligament Registry. All patients who underwent both primary and revision ACLR between January 1, 2005, and December 31, 2023, were included. Patients with multiligament injuries or missing key data were excluded. The prevalence, location, and severity of cartilage lesions assessed intraoperatively at both primary and revision ACLR were reported and compared. Secondarily, potential risk factors for cartilage damage at the time of revision surgery were evaluated using multivariate logistic regression analysis, incorporating available patient and surgical variables from the registry.

**Results:**

A total of 2845 patients were included. The prevalence, size, and severity of cartilage lesions increased markedly across all knee compartments between primary and revision ACLR, which occurred at a median of 2.4 years later. We found 4 independent risk factors for cartilage damage at revision surgery: older age, meniscal and cartilage lesions at primary surgery, and longer time from primary injury to revision ACLR. Each additional month from the initial injury to revision ACLR was associated with a 0.7% increase in the odds of cartilage damage, underscoring a time-dependent degenerative process.

**Conclusion:**

This study showed a clear progression of cartilage degeneration between primary and revision ACLR. Although limited to patients requiring revision surgery, these findings contribute to the broader understanding of posttraumatic joint deterioration and reinforce the need for interdisciplinary approaches to mitigate long-term cartilage damage.

Osteoarthritis (OA) is a growing global health challenge, imposing a substantial burden on patients and health care systems worldwide.^10,33^ Epidemiological data suggest that OA prevalence will continue to rise in the coming years,^23,33^ with the knee being the most commonly affected joint, accounting for approximately 60% of all OA cases.^23^ Among the various causes of knee OA, posttraumatic OA disproportionately affects young and active patients. This subpopulation presents unique challenges in clinical management and contributes significantly to societal costs due to reduced work capacity and long-term disability.^8^

Anterior cruciate ligament (ACL) injuries are common in this population and are strongly associated with an increased risk of developing OA over time.^14^ A key driver of this progression is the presence of cartilage lesions, frequently observed during ACL reconstruction (ACLR), with a reported prevalence ranging from 20% to over 40%, depending on patient characteristics, timing of surgery, and detection method.^9,28^ Cartilage damage may be sustained at the time of the trauma or develop progressively because of altered biomechanics and joint environments after an ACL injury.^6,18,35^ Although ACLR aims to restore knee stability, it does not fully normalize joint loading or halt the biological processes contributing to cartilage degeneration.^17,28,38^ Therefore, understanding the progression of cartilage lesions is critical for improving long-term outcomes and guiding preventive strategies.

This study aimed to describe the prevalence, anatomic distribution, size, and severity of cartilage lesions at the time of both primary and revision ACLR in a unique cohort of patients and to identify risk factors for cartilage lesion progression using data from the Swedish Knee Ligament Registry (SKLR). The hypothesis was that cartilage damage would increase significantly over time and that risk factors for cartilage lesion progression would be associated with the initial injury rather than a new traumatic event.

## Methods

Analysis of the Swedish ACL register was approved by the Regional Ethics Committee of Stockholm (No. 2011/337-31/3). The registry complies with Swedish data security legislation. Participation in the SKLR is voluntary for both patients and surgeons, and no written consent is required for the use of national registry data in Sweden. The study was also approved by the SKLR Steering Committee.

### Study Design and Data Source

This was a retrospective cohort study using prospectively collected data from the SKLR, a nationwide surgical quality registry established in 2005.^2^ The SKLR provides longitudinal surveillance of knee ligament reconstruction procedures across Sweden and includes standardized intraoperative data on graft type, surgical technique, fixation method, meniscal and cartilage abnormalities, and treatment of associated injuries. It also captures patient characteristics, injury and surgery dates, and patient-reported outcome measure scores, including those for the Knee injury and Osteoarthritis Outcome Score^34^ and the EQ-5D questionnaire.

### Study Population

All patients recorded in the SKLR who underwent both primary and revision ACLR between January 1, 2005, and December 31, 2023, were eligible. Exclusion criteria included (1) multiligament injuries involving the posterior cruciate ligament and posterolateral corner, regardless of the treatment, or a medial collateral ligament that was surgically reconstructed; (2) missing data on injury mechanism, graft type, or surgery dates; and (3) missing intraoperative data on cartilage lesion status.

### Outcomes of Interest

Cartilage lesions were assessed intraoperatively at the time of both primary and revision ACLR. Each lesion was categorized by anatomic location (medial/lateral femoral condyle, medial/lateral tibial plateau, trochlea, medial/lateral patella), size (<2 cm^2^ or ≥2 cm^2^), and severity using the International Cartilage Repair Society (ICRS) classification system (grades 1-4). Revision ACLR served as a second-look opportunity, allowing the direct evaluation of cartilage status over time in the same patients.

The primary outcomes included (1) change in the overall prevalence and anatomic distribution of cartilage lesions between primary and revision ACLR, (2) development of new cartilage lesions in knees without any lesions at the time of primary ACLR, and (3) descriptive characterization of lesion severity and size at both time points. Secondary outcomes included the identification of risk factors for cartilage damage at revision surgery, based on patient and surgical variables available from the registry, including age, sex, contralateral ACL injury, time from primary injury to primary surgery, time from primary surgery to revision surgery, time from primary injury to revision surgery, meniscal status and treatment at primary surgery, graft type used in the primary procedure, and chondral status at primary surgery.

### Statistical Analysis

Categorical variables were presented as numbers and percentages; continuous variables were presented as means and standard deviations or medians and interquartile ranges (IQRs) for nonnormally distributed data. The McNemar test was used to compare the prevalence of cartilage lesions between primary and revision ACLR. Univariate logistic regression was used to examine associations between potential predictors and the presence of cartilage lesions at revision ACLR. Multivariate logistic regression was used to confirm the univariate regression results. Among the significant variables identified by univariate regression analysis, temporal variables were mathematically dependent, producing unstable and contradictory coefficients when included simultaneously. Similarly, meniscal lesions (overall, medial/lateral) and meniscal treatment (meniscectomy/repair) overlapped almost completely, as treatment is conditional on lesion presence and morphology. These relationships produced clear multicollinearity in preliminary models, with variance inflation and coefficient reversal. To avoid multicollinearity, the final multivariate model retained only 1 time-dependent variable (time from first injury to revision surgery) and 1 meniscal variable (meniscal lesion at primary surgery), along with age, sex, and cartilage lesion at primary surgery. Odds ratios (ORs) with 95% confidence intervals (CIs) and *P* values were reported. All analyses were conducted using SPSS Statistics (Version 25; IBM). Statistical significance was set at *P* < .05.

## Results

### Patient Characteristics

**Figure 1.**
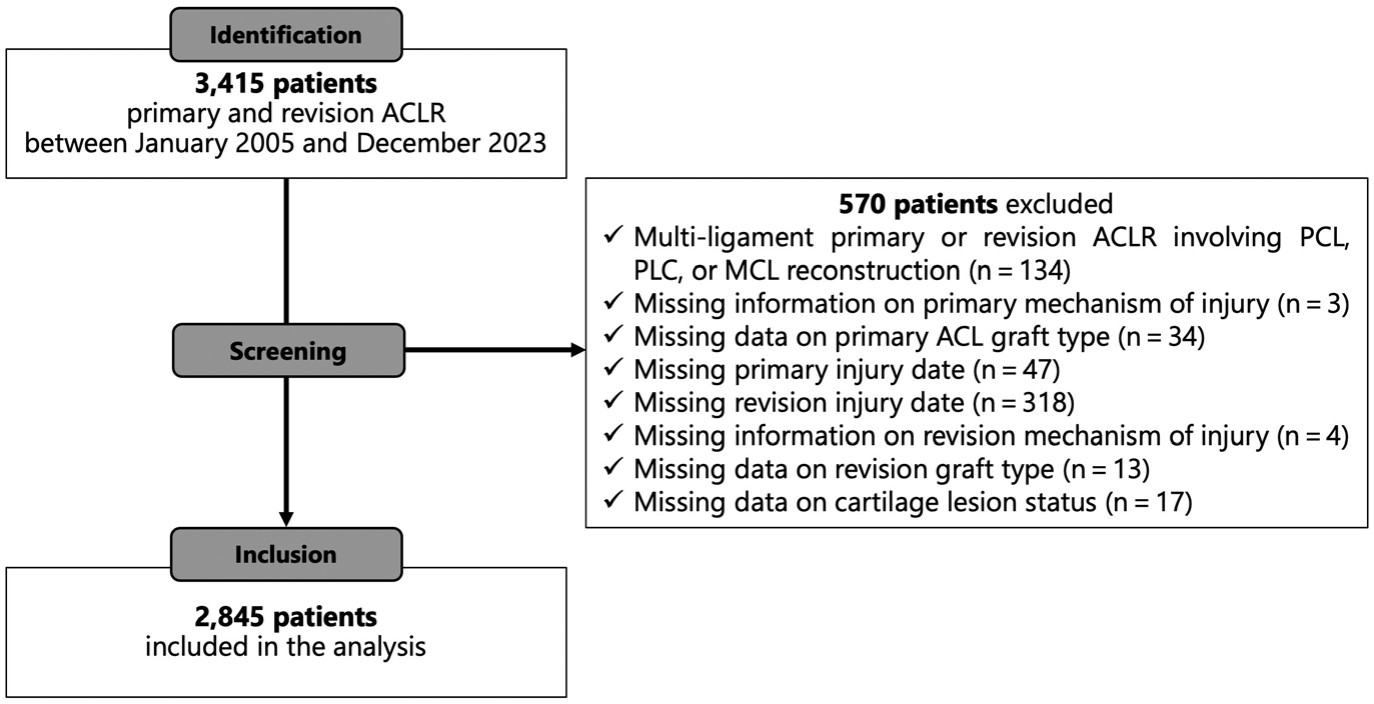
Flowchart of included patients. ACLR, anterior cruciate ligament reconstruction; MCL, medial collateral ligament; PCL, posterior cruciate ligament; PLC, posterolateral corner.

A total of 3415 patients who underwent both primary and revision ACLR between January 1, 2005, and December 31, 2023, were identified in the SKLR. After applying the exclusion criteria, the final cohort consisted of 2845 patients, including 111 with associated medial collateral ligament injuries managed nonsurgically or with suture repair (Figure 1).

The median age at the time of primary surgery was 20.0 years (IQR, 17.0-24.0 years), and the median time from primary ACLR to revision ACLR was 2.4 years (IQR, 1.3-4.5 years). Meniscal lesions were common at both surgical time points, affecting 43% of the patients at primary ACLR and 42% at revision ACLR. Meniscectomy was the most frequently performed meniscal procedure (28% for primary and 26% for revision). The hamstring tendon was the predominant graft choice at primary ACLR (93%), while the bone–patellar tendon–bone was most commonly used at revision ACLR (58%) (Table 1).

**Table 1:** Patient and Surgical Characteristics*^*a*^*

	Value (n = 2845)
Sex	
Female	1289 (45)
Male	1556 (55)
Side	
Right	1457 (51)
Left	1388 (49)
Contralateral ACLR	
No	2708 (95)
Yes	137 (5)
Age at primary surgery, y	20.0 (17.0-24.0)
Time from primary injury to primary surgery, mo	5.3 (3.0-10.2)
Time from primary surgery to revision surgery, y	2.4 (1.3-4.5)
Age at revision surgery, y	23.0 (19.0-28.0)
Time from second injury to revision surgery, mo	8.0 (4.3-16.8)
Meniscal lesions at primary surgery	
Overall	1236 (43)
MM	637 (22)
LM	798 (28)
Untreated meniscal tears	
MM	93 (3)
LM	162 (6)
Meniscal treatment at primary surgery	
Meniscectomy	
Overall	790 (28)
MM	380 (13)
LM	486 (17)
Meniscal repair	
Overall	299 (11)
MM	168 (6)
LM	162 (6)
Graft at primary surgery	
Bone–patellar tendon–bone	143 (5)
Hamstring tendon	2638 (93)
Quadriceps tendon	49 (2)
Other	15 (<1)
Meniscal lesions at revision surgery	
Overall	1180 (42)
MM	745 (26)
LM	634 (22)
Meniscal treatment at revision surgery	
Meniscectomy	
Overall	751 (26)
MM	425 (15)
LM	394 (14)
Meniscal repair	
Overall	408 (14)
MM	256 (9)
LM	193 (7)
Graft at revision surgery	
Bone–patellar tendon–bone	1658 (58)
Hamstring tendon	579 (20)
Quadriceps tendon	455 (16)
Allograft	145 (5)
Other	8 (<1)

a Data are reported as n (%) or median (interquartile range). ACLR, anterior cruciate ligament reconstruction; LM, lateral meniscus; MM, medial meniscus.

### Comparison of Cartilage Lesions Between Primary and Revision ACLR

**Figure 2.**
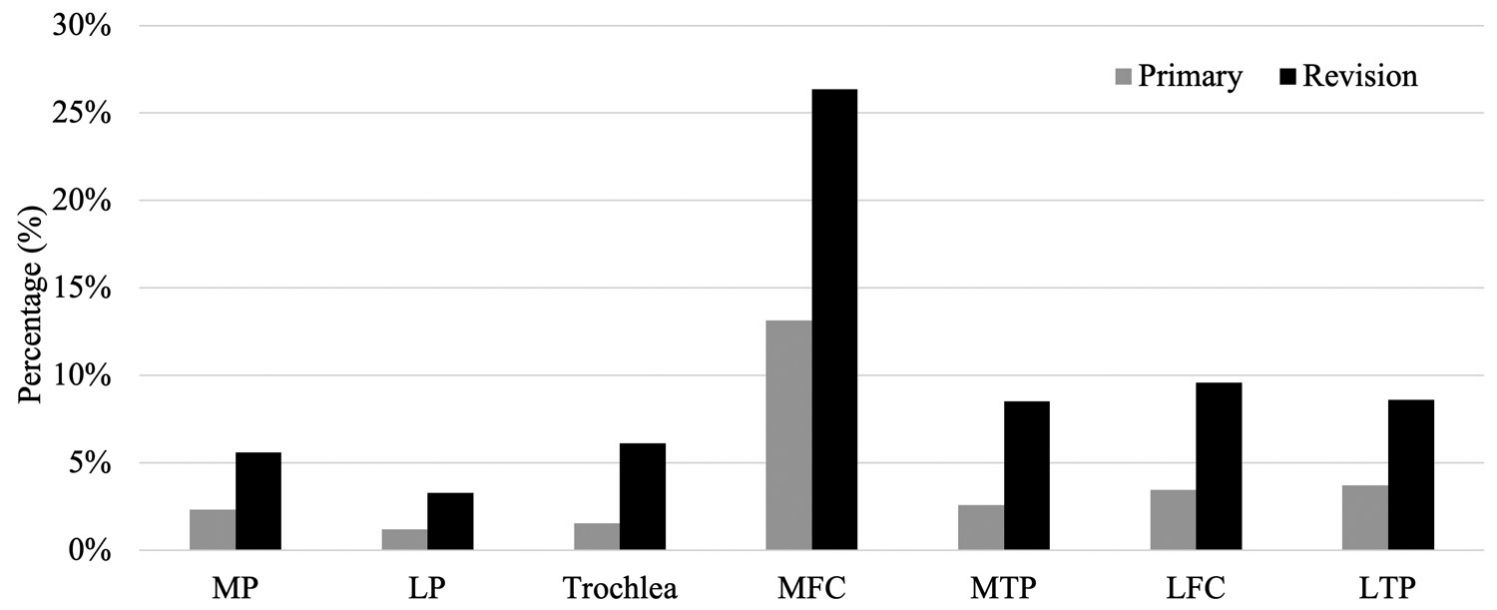
Prevalence of chondral lesions by anatomic site at primary and revision anterior cruciate ligament reconstruction (ACLR). Data are expressed as the percentage of patients with chondral damage at the specified location. Gray bars indicate primary ACLR, whereas black bars indicate revision ACLR.

At the time of primary ACLR, cartilage lesions were documented in 533 patients (19%), while 81% had no documented cartilage involvement. At revision ACLR, the number of patients with cartilage lesions increased to 1025 (36%). All anatomic locations showed a statistically significant increase in lesion prevalence between primary and revision ACLR (*P* < .001 for all). The ORs for lesion progression were as follows: medial patella (OR, 9.642 [95% CI, 5.620-16.543]), lateral patella (OR, 11.687 [95% CI, 5.292-25.809]), trochlea (OR, 8.595 [95% CI, 4.516-16.358]), medial femoral condyle (OR, 5.831 [95% CI, 4.637-7.332]), medial tibial plateau (OR, 9.292 [95% CI, 5.744-15.030]), lateral femoral condyle (OR, 6.149 [95% CI, 3.993-9.470]), and lateral tibial plateau (OR, 8.572 [95% CI, 5.670-12.960]) (Figure 2).

**Figure 3.**
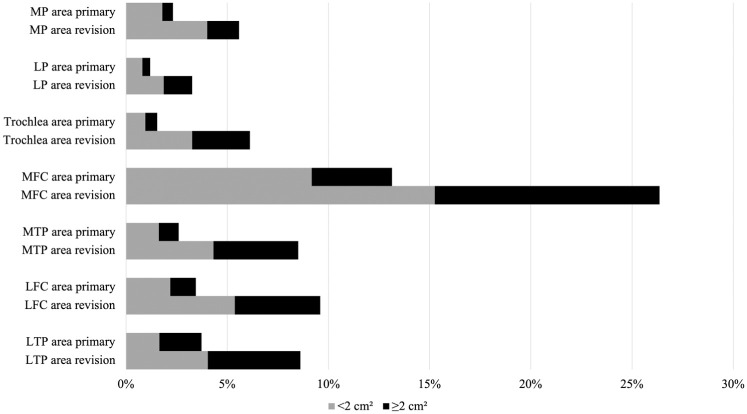
Size of chondral lesions (<2 cm^2^ and ≥2 cm^2^) by anatomic site at primary and revision anterior cruciate ligament reconstruction (ACLR). Data are expressed as the percentage of patients with chondral damage at the specified location. Gray bars represent <2-cm^2^ lesions, and black bars represent ≥2-cm^2^ lesions.

**Figure 4.**
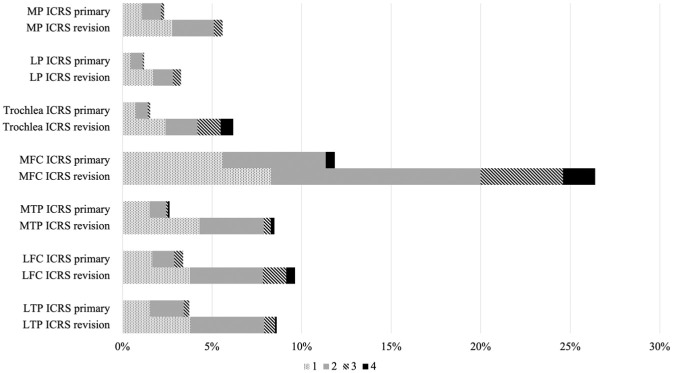
Severity of chondral lesions (International Cartilage Repair Society [ICRS] grades 1-4) by anatomic site at primary and revision anterior cruciate ligament reconstruction (ACLR). Data are expressed as the percentage of patients with chondral damage at the specified location. Dotted white bars represent grade 1, gray bars represent grade 2, diagonally striped bars represent grade 3, and black bars represent grade 4.

At the time of primary ACLR, the medial femoral condyle was the most frequently affected site (13%). Across most sites, smaller lesions (<2 cm^2^) and lower grade cartilage damage (ICRS grades 1-2) were predominant, accounting for 44% to 77% of lesion sizes and 84% to 94% of lesion grades, depending on the location (Table 2 and Figures 3 and 4).

**Table 2:** Cartilage Lesions*^*a*^*

		Lesion Size, cm^2^		ICRS Grade			
	Overall	<2	≥2	1	2	3	4
Primary ACLR							
MP	66 (2)	51 (77)	15 (23)	31 (47)	30 (45)	5 (8)	00 (0)
LP	34 (1)	23 (68)	11 (32)	13 (38)	19 (56)	2 (6)	00 (0)
Trochlea	44 (2)	27 (61)	17 (39)	21 (48)	19 (43)	3 (7)	1 (2)
MFC	374 (13)	261 (70)	113 (30)	157 (42)	161 (43)	42 (11)	14 (4)
MTP	74 (3)	46 (62)	28 (38)	43 (58)	26 (35)	2 (3)	3 (4)
LFC	98 (3)	62 (63)	36 (37)	47 (48)	35 (36)	14 (14)	2 (2)
LTP	106 (4)	47 (44)	59 (56)	44 (42)	53 (50)	9 (8)	00 (0)
Revision ACLR							
MP	159 (6)	114 (72)	45 (28)	79 (50)	66 (42)	13 (8)	1 (1)
LP	93 (3)	53 (57)	40 (43)	49 (53)	31 (33)	12 (13)	1 (1)
Trochlea	174 (6)	93 (53)	81 (47)	68 (39)	51 (29)	36 (21)	19 (11)
MFC	750 (26)	434 (58)	316 (42)	236 (32)	333 (44)	130 (17)	51 (7)
MTP	242 (9)	123 (51)	119 (49)	122 (50)	102 (42)	11 (5)	7 (3)
LFC	273 (10)	153 (56)	120 (44)	107 (39)	116 (42)	37 (14)	13 (5)
LTP	245 (9)	115 (47)	130 (53)	108 (44)	117 (48)	18 (7)	2 (1)

a Data are reported as n (%). ACLR, anterior cruciate ligament reconstruction; ICRS, International Cartilage Repair Society; LFC, lateral femoral condyle; LP, lateral patella; LTP, lateral tibial plateau; MFC, medial femoral condyle; MP, medial patella; MTP, medial tibial plateau.

At the time of revision ACLR, the medial femoral condyle (26%) and lateral femoral condyle (10%) remained the most commonly affected sites. Lesion sizes were more evenly distributed between <2 cm^2^ and ≥2 cm^2^, and there was a notable increase in the proportion of higher grade lesions (ICRS grades 3-4) across all sites (Table 2 and Figures 3 and 4).

### Risk Factors for Cartilage Lesions at Revision ACLR

On univariate analysis, older age, male sex, and all time and meniscal variables were significantly associated with the presence of cartilage lesions in the knee at the time of revision ACLR (Table 3). Neither graft type nor injured side was significant.

**Table 3:** Predictive Factors for Cartilage Lesions at Revision ACLR*^*a*^*

	Univariate		Multivariate	
	OR (95% CI)	*P* Value	OR (95% CI)	*P* Value
Age, y	1.045 (1.034-1.056)	<.001	1.025 (1.014-1.037)	<.001
Sex	1.331 (1.140-1.555)	<.001	1.155 (0.978-1.365)	.090
Time from primary injury to primary surgery, mo	1.009 (1.004-1.013)	<.001		
Time from primary surgery to revision surgery, mo	1.008 (1.006-1.010)	<.001		
Time from primary injury to revision surgery, mo	1.007 (1.006-1.009)	<.001	1.007 (1.005-1.009)	<.001
Meniscal lesion at primary surgery	1.747 (1.496-2.039)	<.001	1.441 (1.220-1.701)	<.001
Medial meniscal lesion at primary surgery	1.934 (1.617-2.314)	<.001		
Lateral meniscal lesion at primary surgery	1.356 (1.146-1.605)	<.001		
Meniscectomy at primary surgery	1.863 (1.575-2.203)	<.001		
Meniscal repair at primary surgery	1.285 (1.006-1.640)	.044		
Chondral lesion at primary surgery	4.837 (3.957-5.912)	<.001	4.085 (3.317-5.030)	<.001

a ACLR, anterior cruciate ligament reconstruction; OR, odds ratio.

Multivariate regression analysis identified 4 independent predictors of cartilage lesions at revision ACLR. The strongest association was the presence of a chondral lesion at primary surgery (OR, 4.085 [95% CI, 3.317-5.030]; *P* < .001), followed by the presence of a meniscal lesion at primary surgery (OR, 1.441 [95% CI, 1.220-1.701]; *P* < .001). Time from the initial injury to revision ACLR was also associated with cartilage damage (OR, 1.007 per month [95% CI, 1.005-1.009]; *P* < .001), and older age at the time of revision ACLR demonstrated a modest cumulative effect (OR, 1.025 per year [95% CI, 1.014-1.037]; *P* < .001).

## Discussion

This large-scale registry-based study demonstrated a clear progression of cartilage degeneration between primary and revision ACLR. Using a second-look design, intraoperative cartilage status was analyzed at both primary and revision ACLR in the same patients. The cartilage lesion burden increased across all sites, with high-grade and large lesions becoming more prevalent over time.

Importantly, multivariate analysis identified 4 independent predictors of cartilage damage at revision ACLR. The age effect was modest but cumulative (OR, 1.025 per year), approximating 28% higher odds per decade, which may reflect age-related cartilage vulnerability and cumulative exposure. The strongest association was observed for patients with cartilage lesions already present at the time of primary surgery, which conferred more than a 4-fold increase in the odds of damage at revision surgery. In addition, time from the initial injury to revision ACLR was significantly associated with cartilage degeneration, with each additional month corresponding to a 0.7% increase in the odds of cartilage damage. Although the monthly effect size appears small, its compounding nature equates to 8.7% higher odds per year and approximately 49% over 5 years, underscoring the clinically meaningful time dependence of chondral deterioration. Previous studies have reported a higher prevalence and severity of cartilage lesions in revision ACLR compared to primary ACLR, often attributing this to the progression of damage over time, cumulative trauma, or surgical failure.^5,25,32^ However, the present study addresses this gap by demonstrating that cartilage deterioration occurs longitudinally, even in knees that have undergone surgical stabilization.

The role of ACLR in preventing posttraumatic OA remains controversial. While surgical reconstruction restores mechanical stability, it does not fully normalize joint biomechanics or halt the biological processes contributing to cartilage degeneration.^22,30^ A national total population study by Nordenvall et al^30^ even suggested that ACLR may be associated with a slightly higher long-term OA risk compared to nonsurgical management. Similarly, a recent systematic review and meta-analysis found comparable radiographic OA prevalence between surgically and nonsurgically treated patients at a median follow-up of 15 years (38% vs 41%, respectively).^21^

The role of the meniscus in knee cartilage deterioration has been widely examined, particularly in ACL deficiency and ACLR.^3,26,31^ Meniscal tears and meniscectomy have been identified as risk factors for cartilage deterioration over time.^3,24,31^ However, most studies, including the present one, did not differentiate patients by lesion pattern or meniscectomy extent, limiting our understanding of the true relationship between meniscal injuries, meniscal treatment, and cartilage damage progression.^36^ The protective role of repair remains unclear, with higher rates of reoperations,^29^ slower return to sport, and less satisfactory knee function reported.^37^ Meniscal status might also introduce selection bias in the present study. Meniscal deficiency is considered a potential cause of ACLR failure, as highlighted by the International Meniscus Reconstruction Experts Forum recommendation to perform meniscal transplantation in revision ACLR.^12^ Our cohort might have a higher prevalence of primary meniscectomy than the general ACLR population, an intrinsic limitation that allows evaluating the knee through second-look arthroscopy.

Although a meniscal injury could represent a relevant contributor to cartilage degeneration,^5,31^ an ACL injury itself alters joint loading patterns and initiates inflammatory cascades that may accelerate chondral damage.^1,16-18^ The findings of this study suggest that these processes continue over time between primary and revision surgery, even after a surgical intervention, and may not be adequately addressed by current treatment strategies, although subsequent injury events and continued participation in pivoting sports could also contribute.

Regarding graft type, our findings align with the literature, which shows no clear influence of graft choice.^4^ In our cohort, the number of bone–patellar tendon–bone grafts was relatively small, which limits the strength of any graft-specific comparisons. Some studies suggest a higher risk of OA with patellar tendon grafts likely because of patellofemoral biomechanical changes.^24^

Understanding why an ACL injury frequently leads to OA remains one of the major challenges currently faced by researchers. At present, surgical techniques allow patients to return to sport with acceptable rates of failure, particularly when combined with appropriate rehabilitation^15,19^ and a consideration of concomitant factors such as meniscal lesions or anterolateral complex injuries.^11,13^ What is still lacking, however, is the ability to prevent the development of posttraumatic OA, which occurs in up to 50% of patients after an ACL injury and represents a considerable public health burden.^8,22^ Patients affected by posttraumatic OA are typically young, physically active persons, a scenario usually described as “a young patient with an old knee.”^22^ This condition not only affects quality of life but also imposes substantial social and economic costs through increased outpatient visits, work absenteeism, joint replacement procedures, and the potential onset of comorbidities associated with a more sedentary lifestyle.^7,20,27^

An ACL injury should therefore be recognized not only as an orthopaedic issue but also as a sentinel event in a systemic degenerative process that current surgical strategies cannot fully address. The progression of cartilage damage in the knee mirrors similar patterns in other joints after trauma, suggesting a shared biological pathway that transcends surgical boundaries.^18^ With these considerations in mind, a shift in ACL research from refining surgical techniques to investigating the biomechanical and biological reasons for posttraumatic OA should be advocated. Surgeons alone cannot solve the challenge of posttraumatic OA. To truly address this complex and progressive condition, ACL research must expand beyond surgical refinement and embrace a deeply interdisciplinary approach. Collaboration across molecular biology, immunology, biomechanics, rehabilitation, regenerative medicine, and data science is essential not only to develop novel diagnostics and systemic therapies but also to fundamentally understand and interrupt the biological and mechanical cascades that drive joint degeneration after trauma.

### Limitations

This study has several limitations. As with all large-scale registry studies, there is a risk of missing or inconsistently reported data, which may introduce information bias and affect the robustness of certain analyses. Additionally, the observational nature of the study means that patients were not randomized, and the number of available variables is limited compared to prospective designs. This introduces potential selection bias and may leave residual confounding factors unaccounted for in risk analysis. Another important limitation stems from the inclusion criteria, which required patients to have undergone both primary and revision ACLR. This inherently selects for those with graft failure or recurrent instability and may overestimate the prevalence and severity of cartilage lesions because of the presence of a second traumatic event or chronic joint dysfunction. However, to ethically justify a second arthroscopic assessment of cartilage status, revision surgery provides the only feasible context. This methodological constraint is shared by all studies employing second-look designs. Lastly, the registry provides limited information on patient-specific anthropometric factors (such as posterior tibial slope) and meniscal tear patterns, which might have influenced the prevalence of ACL retears and chondral damage, potentially introducing bias. Other potentially relevant factors, such as activity level between procedures, body mass index, and lower limb alignment, were not available in the registry. Advanced imaging modalities (eg, T2 mapping magnetic resonance imaging) may help to characterize early cartilage changes in future studies.

## Conclusion

This study showed a clear progression of cartilage degeneration between primary and revision ACLR. Although limited to patients requiring revision surgery, these findings contribute to the broader understanding of posttraumatic joint deterioration and reinforce the need for interdisciplinary approaches to mitigate long-term cartilage damage.
